# *Bergenia ciliate*–Mediated Mixed-Phase Synthesis and Characterization of Silver-Copper Oxide Nanocomposite for Environmental and Biological Applications

**DOI:** 10.3390/ma14206085

**Published:** 2021-10-14

**Authors:** Fazal Ur Rehman, Rashid Mahmood, Manel Ben Ali, Amor Hedfi, Mohammed Almalki, Amine Mezni, Wajid Rehman, Sirajul Haq, Humma Afsar

**Affiliations:** 1 Department of Chemistry, University of Azad Jammu and Kashmir, Muzffarabad 13100, Pakistan; fazal.rehman@ajku.edu.pk (F.U.R.); rashid.mehmood@jku.edu.pk (R.M.); humma.afsar.mphil@ajku.edu.pk (H.A.); 2 Department of Biology, College of Sciences, Taif University, P.O. Box 11099, Taif 21944, Saudi Arabia; mjbinali@tu.edu.sa (M.B.A.); o.zaied@tu.edu.sa (A.H.); almalki.m@tu.edu.sa (M.A.); 3 Department of Chemistry, College of Science, Taif University, P.O. Box 11099, Taif 21944, Saudi Arabia; a.rachid@tu.edu.sa; 4 Department of Chemistry, Hazara University, Mansehra 21300, Pakistan; wajid757@hu.edu.pk

**Keywords:** copper oxide, silver, ascorbic acid, antioxidant, application

## Abstract

*Bergenia ciliate* (*B. ciliate*) leaf extract was used as a reducing and stabilizing agent for the synthesis of silver-copper oxide nanocomposite (Ag-CuO NC). Scanning and transmission electron microscopies (SEM and TEM) were used to examine the structural morphology, and the average particle size was determined to be 47.65 nm. The phase confirmation and crystalline structure were examined through the X-ray diffraction (XRD) technique, where cubic and monoclinic geometries were assigned to Ag and CuO. The energy dispersive X-ray (EDX), Fourier transform infrared (FTIR) and ultra-violet and visible (UV-Visible) spectroscopies were operated to analyse the elemental composition, functional groups and light absorption phenomena of the Ag-CuO NC. Under the full light spectrum, the photodegradation of Rhodamine 6G was recorded, and 99.42 percent of the dye degraded in 80 min. The Agar well diffusion method was followed to perform antibacterial activity against selected pathogens, and the activity was found to increase with increasing concentration of Ag-CuO NC. The ABTS free radical scavenging activity suggests that the activity of Ag-CuO NC is higher than ascorbic acid.

## 1. Introduction

Organic dyes are extensively utilized in photo-electrochemical cells, light-harvesting arrays, agricultural research, the medical field, food technology, paper production, and the leather and textile industries and are thus released into the aquatic environment. Due to toxicity and stability, these dyes cause serious health problems and environmental pollution [1]. Multi-drug resistant microbes in parasites, viruses, fungi, and bacteria are increasing microbial infections. By applying several techniques, such as type IV secretion systems, multidrug efflux pumps, transposons, integrons, and R plasmids, bacteria can resist antibiotic agents. Biofilm production is an another problem caused by microorganisms; it leads to bacterial resistance to desiccation and starvation conditions [2,3]. Environmental pollution and microbial contamination are significant problems that generate widespread concern. Thus, an efficient and reliable solution is required to protect aquatic and terrestrial life.

Semiconductor nanocomposites play a great role in removing industrial pollutants. To treat waste water, various methods are used, including advanced oxidation methods, surface phenomena, catalytic reduction, electro-chemical corrosion, UV-light degradation, and activated carbon sorption [4]. Photocatalysis is a green method, used for energy issues and environmental remediation due to its low cost and eco-friendly nature [5]. Due to photodegradation capability, nanocomposites of metal oxides have attained a valuable position and attracted great attention in green chemistry as well as the medical field and show superior antimicrobial activity [6]. Copper oxide is widely available, more eco-friendly, stable, less toxic, cheaper, and has shown enhanced antibacterial activity [7]. Silver nanoparticles have demonstrated antibacterial activity against Gram-positive and Gram-negative bacteria [8,9]. Because of their large surface area, silver nanoparticles easily interact with bacterial membranes, showing their activity. Copper- and silver-based nanocomposites have had wide application in different areas, such as fungicidal uses, textile industries, water-based systems, the health industry, biomedical applications, wound dressing, medicine, and the food and cosmetics industries [6,10]. Based on the literature, it has been observed that plant-mediated synthesis of Ag-CuO NC has yet to be reported. Moreover, *B. ciliate* is composed of phytochemicals like Bergenin, Tannic acid, Gallic acid, Stigmesterol, -Sitosterol, Catechin, (+)-Afzelechin, 1.8-cineole, Isovalaric acid, (+)-(6S)-parasorbic acid, Arbutin, -eudesmol, 3-methyl-2-buten-1-ol, (Z)-asarone, and Terpin and is expected to be an efficient reducing and capping agent. It is expected that due to the synergistic effect of silver and copper oxide, synthesized Ag-CuO NPs will exhibit enhanced photocatalytic activity along with high biological potency.

In this study, Ag-CuO NC was synthesized by the green method using leaf extracts, and its physiochemical properties were determined through FTIR, SEM, TEM, XRD, and UV-Visible spectroscopy. The antibacterial, antioxidant, and photocatalytic efficacy of Ag-CuO NC was examined for selected species of bacteria, ABTS free radicals, and Rhodamine 6G, respectively.

## 2. Materials and Methods

### 2.1. Chemicals Used

In the current experiment, analytical grade chemicals were used, including silver nitrate, copper sulphate, ethanol, rhodamine 6G, and Agar nutrition. All of the required solutions were made using distilled water.

### 2.2. Preparation of B. ciliata Leaf Extract

A total of 20 g of *B. ciliata* leaves was boiled in 200 mL of distilled water at 70 °C to make the aqueous extract. The clear extract was obtained after filtering the aqueous extract twice using Whatmann No. 1 filter (MilliporeSigma, MO, USA) paper for 15 min. For the production of nanomaterials, an aqueous leaf extract was employed as a reducing agent.

### 2.3. Synthesis of Ag-CuO NC

For the typical synthesis of Ag-CuO NC, a 5 mM solution of AgNO_3_ was prepared, and from this, 60 mL was mixed with 30 mL of *B. ciliate* extract, with constant heating and stirring for 45 min. The colloidal suspension containing Ag-NPs was marked as solution A. Similarly, another 5 mM of solution was prepared by dissolving the appropriate amount of CuSO_4_ and was mixed with extract with ratio of 2:1, with constant heating and stirring for 45 min. The gel formed was aged for 4 h and marked as solution B. Then, both the solutions were slowly mixed with vigorous stirring at 80 °C for 5 h and were then aged for 24 h. The obtained solid product was washed thrice with deionized water, followed by dehydration in an electric oven at 150 °C and storage in an air-tight polyethylene bottle.

### 2.4. Instrumentation 

The crystallite size of the Ag-CuO NPs was calculated using the Debye-Scherrer equation, and the crystalline nature was investigated using the XRD model Philips X’Pert. The microstructure and surface topology were studied using SEM Model JEOL JSM-5600LV, Tokyo, Japan and Hitachi HT7800 transmittance electron microscope (TEM) Cleveland, TN, USA. To validate the sample’s composition, an energy-dispersive X-ray (EDX) examination was performed with a JEOL JSM-5600LV, Oxfordshire, UK paired with a scanning electron microscope (SEM). The light absorption phenomena were studied using Shimadzu (UV-800) (Thermo Fisher Scientific Waltham, MA, USA) spectroscopic analysis (band gap was calculated through Tauc’s plot), while surface functional moieties were explored using FTIR model Nicolet 560, operated in the range of 4000–400 cm^−1^.

### 2.5. Photocatalytic Assay

The photocatalytic efficacy of Ag-CuO NC was evaluated during the degradation of Rhodamine 6G in the presence full light spectrum in the month of June between 11:00 am and 3:00 pm. For the typical photochemical reaction, 25 mg of the catalyst was added to the reaction vessel containing 70 mL Rhodamine 6G solution and was stirred in darkness for 30 min. Afterward, the reaction mixture was illuminated under full light spectrum, and the UV analysis was carried out after a specific time interval. The decrease in the absorbance maxima with the passage of time suggests the degradation of Rhodamine 6G. The stability of Ag-CuO NC was checked under the same experimental conditions by adding a fresh dye solution five times into the reactor containing the already used catalyst.

### 2.6. Antibacterial Assay

The antibacterial activity of Ag-CuO NC was tested using the Agar well diffusion technique against *E. coli* and *S. aureus*. The plates were prepared by mixing Agar nutrients in deionized water and left to solidify naturally at room temperature. The overnight growing bacterial culture was applied to the medium, and the wells were drilled with a polystyrene tip. The stock suspension was prepared by ultrasonic dispersion of 5, 15, 25, and 50 mg of Ag-CuO NC in 5 mL deionized water, and 100 μL was added into each well. After 24 h of incubation at 37 °C, the zone of inhibition was measured in millimetres (mm) as the activity of Ag-CuO NC.

### 2.7. Antioxidant Assay

First, the ABTS^•+^ was generated by combining 5 mM potassium persulphate and 14 mM ABTS (1:1 (*v/v*) and 12:8 (*w/w*)) in darkness for 16 h, and the absorbance of the solution was measured at 734 nm. The stock suspensions of Ag-CuO NC were prepared by ultrasonic dispersion of 5, 25, 50, 75, 100, and 200 μg in 1 mL for 30 min at room temperature. Then, 0.2 mL of Ag-CuO NC suspension and 0.15 mL of ABTS^•+^ solution were mixed and aged for 30 min and then subjected to UV analysis; the absorbance was measured at 734 nm. Equation (1) was used to calculate the percent ABTS free radical inhibition activity, where *Ai* is the absorbance of the sample and *Ao* is the absorbance of the control.

(1) $$\%RSA=\left[\left(\frac{A_{o}+A_{i}}{A_{o}}\right)\right]\times \;100$$

### 2.8. Statistical Measurements

The variance (S^2^), standard deviation (S), and Pearson correlation (*p* = 0.05) for the obtained data was calculated at the 95% confidence interval. All the analyses were carried out using Microsoft Excel 2013 (Microsoft office 2013, Las Vegas, NV, USA).

## 3. Results and Discussion

### 3.1. Physicochemical Study

#### 3.1.1. SEM Analysis

**Figure 1:**
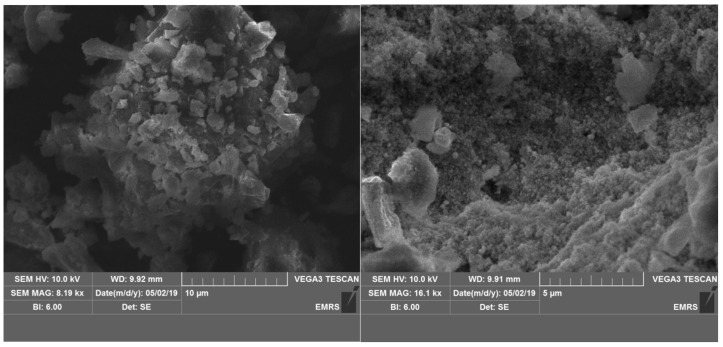
Low and high magnified SEM micrographs of Ag-CuO NC.

The low and high magnified SEM micrographs of Ag-CuO NC, as shown in Figure 1 demonstrate that the background dense solid structure was generated due to particle agglomeration. The prearrangement of the particles is not compact, and cavities with varied sizes are formed due to loss arrangement of the particles. However, several individual particles with different shapes and sizes are also seen on the surface of the solid structure. Upon increasing the magnification, the samples seem to be porous in nature.

#### 3.1.2. TEM Analysis

**Figure 2:**
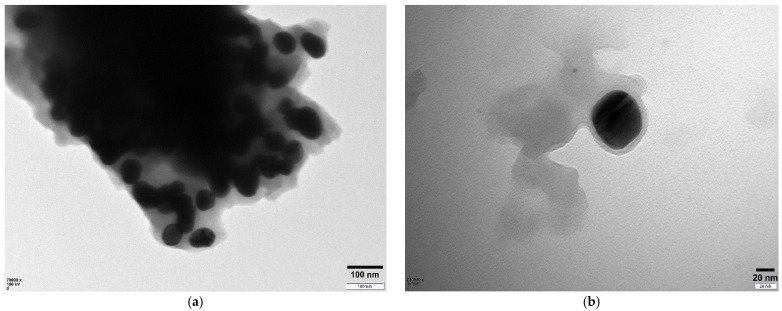
Low (**a**) and high (**b**) magnified TEM micrographs of Ag-CuO NC.

The morphology of Ag-CuO NC was investigated through TEM analysis, and the resultant micrographs at low and high magnifications are given in Figure 2a,b. Similar to the SEM analysis, the TEM analysis at low magnification shows that several particles are closely fused together, leading to the vanishing of particle boundaries and thus to the formation of a dense network of particles. It was also observed that the larger particles with asymmetrical morphology were formed by the fusion of two or three particles. Many individual particles with well-defined boundaries are seen in the sample, which are nearly spherical/oval in shape but with different sizes. A nearly oval-shaped individual particle with a smooth surface is shown in the low magnified TEM image (Figure 2b), and the particle size measured though ImageJ software ranges between 54.77 and 79.05 nm, with an average size of 63.24 nm.

#### 3.1.3. EDX Analysis

**Figure 3:**
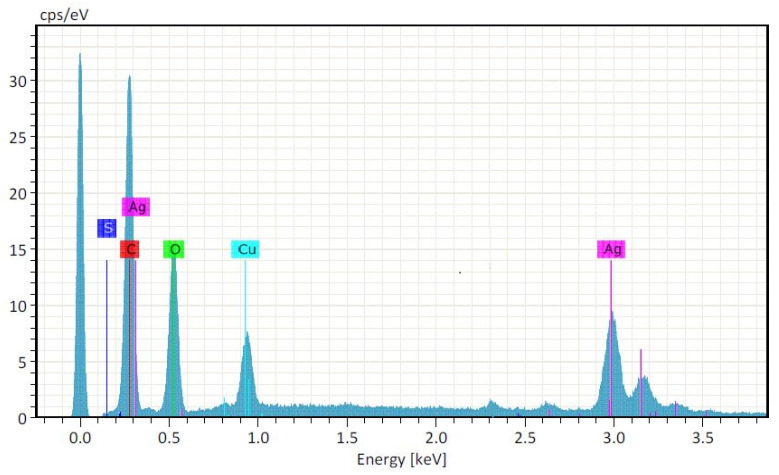
Elemental composition of Ag-CuO NC depicted in EDX spectrum.

The elemental composition and percentage purity of Ag-CuO NC was explored through EDX (Figure 3), and the spectrum possessed strong signals for Ag (at 0.28 and 2.9 keV) and Cu (at 0.91 keV). Three weak signals were also seen in the EDX spectrum, attributed to C, O, and S, where C and S could be due to the X-ray emission of the phytochemicals, as plant leaves were used for the synthesis of the sample [11]. The weight percentage of the detected elements Ag, Cu, O, C, and S were 42.74, 25.16, 20.98, 10.51, and 0.60, respectively.

#### 3.1.4. XRD Analysis

**Figure 4:**
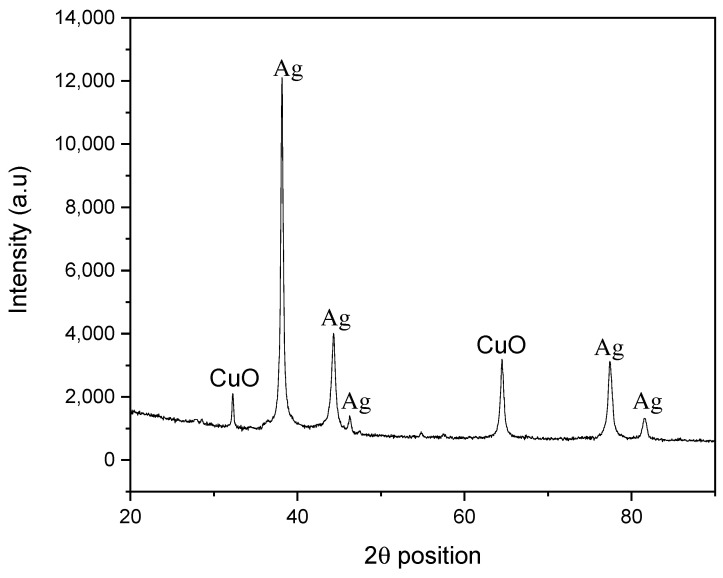
X-ray diffractogram of Ag-CuO NC.

The X-ray diffraction profile (Figure 4) of the synthesized Ag-CuO NC exhibited sharp, intense Bragg’s reflections at 32.83 (−110), 38.27 (111), 44.38 (200), 46.36 (−112), 64.51 (220), 77.55 (311), and 81.51 (222) 2-theta positions, suggesting the formation of a highly crystalline sample. The bands at 2θ angles were 38.27, 44.38, 64.52, 77.55, and 81.51, corresponding to (111), (200), (220), (311), and (222) planes (JCPDS Card No. 00-004-0783), respectively, attributed to the cubic crystal structure of Ag with a space group and space number of Fm3m and 225, respectively. The reflections at the 2θ position with corresponding hkl planes were 32.83 (−110) and 46.36 (−112), matched with the JCPDS card No. 00-045-0937, attributed to the monoclinic crystal shape of CuO, with a space group of C2/c and a space number of 15. According Bragg’s equation, the distance between the planes increases with a reduction of Sin θ (nλ = 2dSinθ). The reduced angles for CuO were attributed to the deposition of Ag on the surface of CuO and the smaller amount of CuO, as was also evident from the EDX analysis [12]. The separate set of diffraction bands for both Ag and CuO suggest the formation of a heterojunction, with different crystalline natures and crystallite geometry.

#### 3.1.5. UV-Visible Analysis

**Figure 5:**
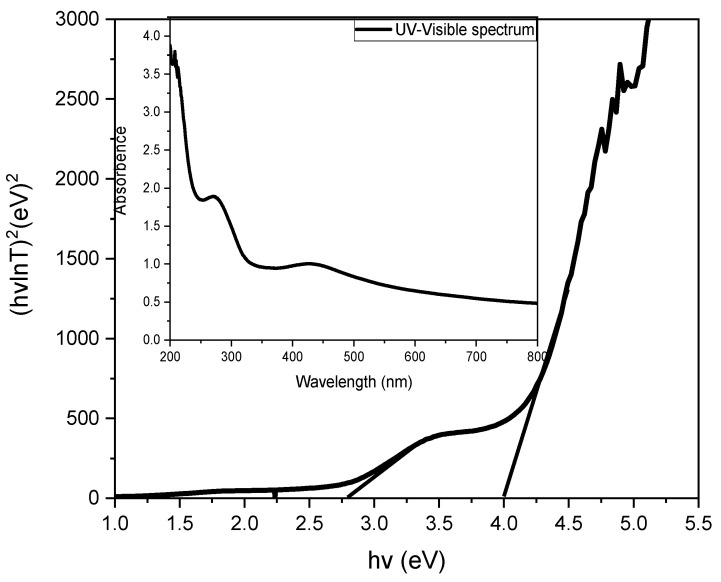
Tauc plot (inset: UV-Visible spectrum) of Ag-CuO NC.

The UV-Visible absorption of the synthesized Ag-CuO NPs was recorded in the range of 200–800 nm, as shown in Figure 5. The absorption band at 271.82 nm was attributed to the charge transfer transition from O^2−^ and Cu^2+^ [13], which confirms the existence of CuO in the sample, whereas the broad absorption peak cantered at 430.09 nm is assigned to the metallic silver nanoparticle [14]. The presence of two absorption bands suggest the formation of an Ag-CuO heterojunction, which is also confirmed by XRD. The band gap energies were determined through the Tauc relation and were found to be 3.96 and 2.78 eV for CuO and Ag, respectively [7].

#### 3.1.6. FTIR Analysis

**Figure 6:**
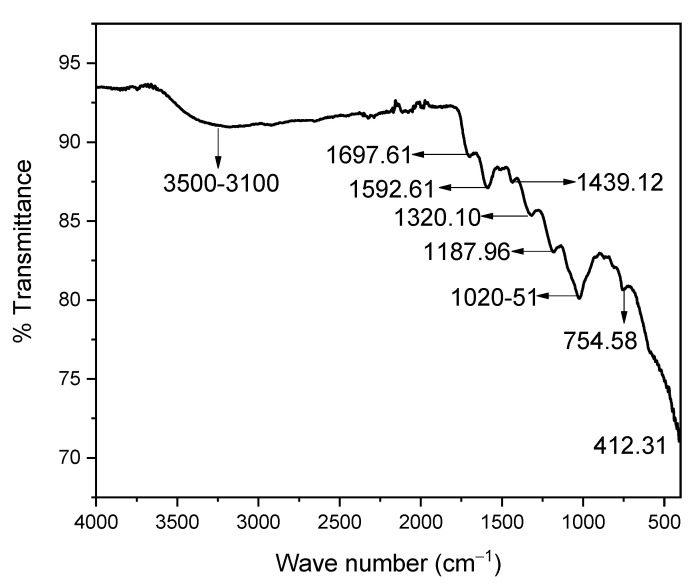
FTIR spectrum of Ag-CuO NC.

The FTIR analysis of Ag-CuO NC in the KBr pellet was performed in the region of 4000 to 400 cm^−1^ (Figure 6), with the stretching of the hydroxyl moiety attributed to the less intense wide band centred at 3367 and 1697.61 cm^−1^ [15]. The two peaks at 1592.61 and 1439.12 cm^−1^ were assigned to carbon-carbon (aromatic carbons) and carbon-hydrogen functionalities, which due to the organic sources (plant leaf extract), were used during synthesis [16]. The peak at 1320.10 cm^−1^ might be due to the presence of NO_3_, as silver nitrate was used as a precursor. The peaks at 1187.96 and 1020.51 cm^−1^ were attributed to oxygen-copper-oxygen and copper-oxygen-copper stretching, respectively [17]. The peak appearing at 754.58 cm^−1^ is due to stretching of the Cu-O with monoclinic geometry [18]. The observed vibration band at 412.31 cm^−1^ is due to the nano-sized silver with cubic geometry [19].

### 3.2. Photocatalytic Study

**Figure 7:**
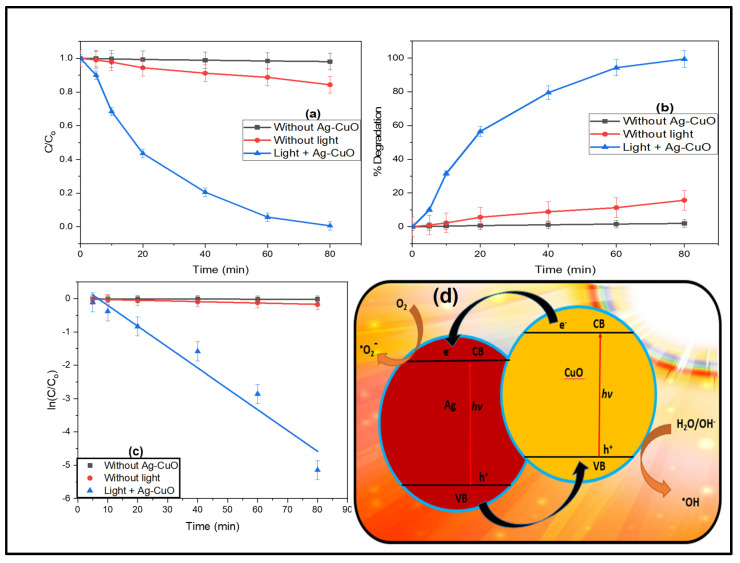
Photocatalytic experiment parameters: (**a**) degradation profile, (**b**) percentage degradation profile, (**c**) kinetic isotherm, and (**d**) electron excitation and hole creation mechanism.

The photocatalytic degradation of organic dyes was one of the important methods used to check the photocatalytic activity of Ag-CuO NC under the full light spectrum, as shown in Figure 7. In the presence of a full light range, photocatalytic degradation of Rhodamine 6G was examined using Ag-CuO NC. The reaction mixture was stirred in the dark for 30 min before the light exposure. After exposure to light, the decrease in the absorbance of Rhodamine 6G was examined in order to check the process of degradation as a function of time. Equations (2) and (3) were used to calculate the percentage of degradation of dye and reaction rate constant, respectively, where C_o_ was the initial concentration of Rhodamine 6G and C_t_ was the concentration of Rhodamine 6G after exposure to light [6]. The self-photolysis experiment was performed with the addition of a catalyst, and only 1.93 percent was degraded, with degradation rate of 0.2353 × 10^−3^/min. The experiment was also performed in the absence of light, and after 80 min, a 15.71% decrease was seen in the concentration of the dye. This decrease is due to the adsorption of dye molecules on the surface of Ag-CuO, with a transfer rate of 0.204 × 10^−2^/min. After the basic analysis, the photocatalytic experiment was carried out in the presence of both light and the catalyst. The percentage degradation of Rhodamine 6G was found to be 99.42% in the time duration of 80 min, whereas the determined degradation rate constant was 0.6261 × 10^−1^ min^−1^. The stability of the catalyst is very important in term of its reusability and has great environmental and economic importance. The Ag-CuO NC was reutilized under the current experimental conditions five times, and after every 80 min, a fresh solution was added to the reactor. The same process was repeated five times, and the percent degradation was found to be 99.42, 98.63, 96.57, 92.46, and 87.93% during the 1st, 2nd, 3rd, 4th, and 5th round respectively. This shows the stability of the synthesized Ag-CuO NC, which can be used for multiple-step degradation of organic pollutants. The photocatalytic efficacy of the nanocatalyst reported in the present research was compared to those reported in the literature, as shown in Table 1.

(2) $$\%\;degradation=\left[\frac{\left(C_{o}-C_{i}\right)}{C_{o}}\right]\times \;100$$

(3) $$ln\left(\frac{C}{C_{o}}\right)=-kt$$

Photocatalytic degradation of Rhodamine 6G is done by using Ag-CuO NC, where the electrons present in the valence band Ag-CuO NC, by illuminating with the help of light are excited towards conduction band. Due to excitation of electrons from valence to conduction band, holes produced in the valence band. Superoxide radical is formed when dissolved oxygen is being reacted by the excited electron which results to the production of hydroxide radicals. Hydroxide radicals were also produced when water molecules reacted with created holes. Thus, a strong oxidizing agent in the form of ·OH radical formed for the degradation of Rhodamine 6G. Because of the combined action of ·OH as a highly oxidizing agent and h^+^ ion, mineralization of organic pollutant occurred. Ag^+^ ions to Ag reduction deplete electrons, which increase the separation of holes/electrons and reduce their recombination. Photo-generated electrons transfer adsorbed oxygen on the surface of Ag nanoparticles and produced O^2−^ species. These O^2−^ take part in the process of photocatalysis and also reduce Ag^+^ ions which results in the formation of Ag^o^ particles on surface [26]. 

### 3.3. Antibacterial Activity

**Figure 8:**
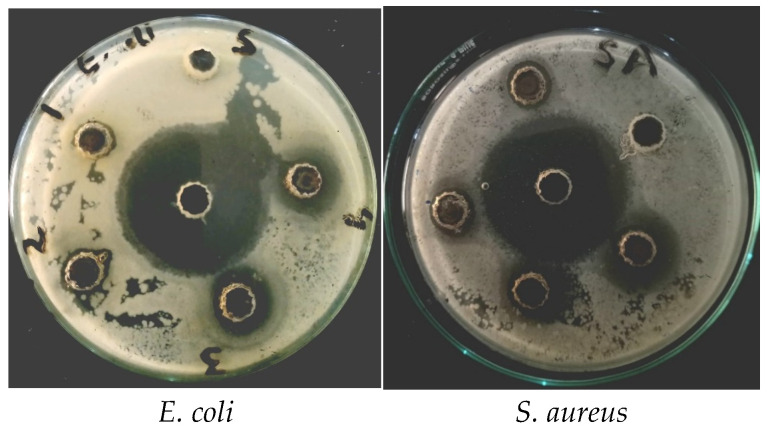
Pictorial representation of bactericidal activity of Ag-CuO NC against *E. coli* and *S. aureus*.

The antibacterial activity of Ag-CuO NC was evaluated against *E. coli* and *S. aureus* using Agar well diffusion method and the obtained experimental photographs are shown in Figure 8. The results shows that the activity of Ag-CuO NC against both microorganism was gradually increased by increasing sample concentration on the well, which may be due to the larger of Ag-CuO particles accumulate on the bacterial surface and penetrate inside the cell. The results also demonstrate that the activity of the Ag-CuO NC was less than the standard drug and the solvent shows no activity. The activity of the nanocomposite are due to the metal cation (Ag^+^ and Cu^2+^) release in aqueous solution, which bind with the thiol group of the bacterial protein and rapture the outer membrane and as a results cytoplasmic fluid leaks outside led to the death of bacteria [27,28].

The slightly higher action of Ag-CuO NC against *E. coli* as compared *S. aureus* are because of the differences in cell wall composition and surface charge of both species. The Gram-negative bacteria’s cell walls have a significant negative charge due to the presence of lipopolysaccharide and phospholipid, thus large number of the metal cation strongly attached on the surface of *E. coli* and cause serious damage to bacterial cell [29]. On the other hand, the surface of Gram-positive bacteria as a partial negative charge due to teichoic acid where interaction between the surface charge and metal cation are weak [30]. Furthermore, the cell wall of Gram-negative bacteria contains a few fragile layers of peptidoglycan, whereas the cell wall of Gram-positive bacteria has numerous strong peptidoglycan layers that give additional strength and resistance to the incoming penetrating agent [31].

### 3.4. Antioxidant Activity

The antioxidant response of Ag-CuO NC was evaluated in methanolic medium to stabilized the free radical (ABTS^•+^). The dose dependent antioxidant study of the Ag-CuO NC indicates that the activity of antioxidant was gradually increases with the increasing concentration as shown in Table 2 and percentage radical scavenging activity was determined by the reaction of ABTS^•+^ and different concentration of the synthesized nanocomposite. The significant potency at higher concentration of the sample are might be due to the larger number of antioxidant that can stabilized more free radicals. The antioxidant activity can also be measure in term of IC_50_ value that can neutralized 50 percent of the free radicals [32]. The IC_50_ value for the Ag-CuO NC is 126.60 µg·mL^−1^ whereas those for the ascorbic acid used as standard is 131.21 µg·mL^−1^, proposed that the antioxidant activity of the synthesized nanocomposite is higher than ascorbic acid [33,34].

## 4. Conclusions

The aim of this study was to synthesize Ag-CuO NC for environmental and biological applications. A hybrid Ag-CuO NC was synthesized through an eco-friendly process using *B. ciliate* leaf extract. The hybrid nature of Ag-CuO NC was confirmed through XRD, EDX, FTIR, and UV-Visible spectroscopy, where independent peaks were observed for Ag and CuO. The cubic and monoclinic geometric shapes were reported with band gap energies of 2.78 and 3.96 eV for Ag and CuO, respectively. The significantly enhanced photocatalytic, antibacterial, and antioxidant activities of Ag-CuO NC were due to the synergistic effect of both counterparts.

## Figures and Tables

**Table 1:** Comparison of the photocatalytic efficacy of different nanocatalysts against rhodamine 6G.

S.No.	Nanocatalysts	Time (min)	Percentage Degradation	References
1	SrCrO_4_/TiO_2_ oxides	360	95	[20]
2	n-TiO_2_	180	11	[21]
3	ZnTiO_3_	180	82	[22]
4	ZnO and TiO_2_	90	71.41; 86.02	[23]
5	Ag–TiO_2_	60	96.57	[24]
6	Zn_2_SnO_4_	90	91	[25]
7	Ag-CuO	80	99.42	Present work

**Table 2:** Antioxidant activity of Ag-CuO NC against ABTS free radicals at different concentrations.

Samples	Concentrations (μg·mL^−1^)	%RSA	IC_50_ Value (μg·mL^−1^)	Variance (S^2^)	Standard Deviation (S)	Correlation b/w the Obtained Data (*p* = 0.05)
Ag-CuO NC	5	41.08	126.60	1.29	1.13	0.010632
	25	49.38				
	50	61.57				
	75	76.63				
	100	85.03				
	200	96.88				
Ascorbic Acid	5	39.1	131.21	1.31	1.14	
	25	49.05				
	50	59.13				
	75	71.49				
	100	81.74				
	200	94.62				

## Data Availability

All the data is available within the manuscript.
