# Correction: Rehman et al. *Bergenia ciliate*–Mediated Mixed-Phase Synthesis and Characterization of Silver-Copper Oxide Nanocomposite for Environmental and Biological Applications. *Materials* 2021, *14*, 6085

**DOI:** 10.3390/ma19071467

**Published:** 2026-04-07

**Authors:** Fazal Ur Rehman, Rashid Mahmood, Manel Ben Ali, Amor Hedfi, Mohammed Almalki, Amine Mezni, Wajid Rehman, Sirajul Haq, Humma Afsar

**Affiliations:** 1 Department of Chemistry, University of Azad Jammu and Kashmir, Muzffarabad 13100, Pakistan; fazal.rehman@ajku.edu.pk (F.U.R.); rashid.mehmood@jku.edu.pk (R.M.); humma.afsar.mphil@ajku.edu.pk (H.A.); 2 Department of Biology, College of Sciences, Taif University, P.O. Box 11099, Taif 21944, Saudi Arabia; mjbinali@tu.edu.sa (M.B.A.); o.zaied@tu.edu.sa (A.H.);; 3 Department of Chemistry, College of Science, Taif University, P.O. Box 11099, Taif 21944, Saudi Arabia; a.rachid@tu.edu.sa; 4 Department of Chemistry, Hazara University, Mansehra 21300, Pakistan; wajid757@hu.edu.pk

In the original publication [1], there was a mistake in Figure 8 as published. The corrected Figure 8 is shown below. The authors state that the scientific conclusions are unaffected. This correction was approved by the Academic Editor. The original publication has also been updated.

## Figures and Tables

**Figure 8:**
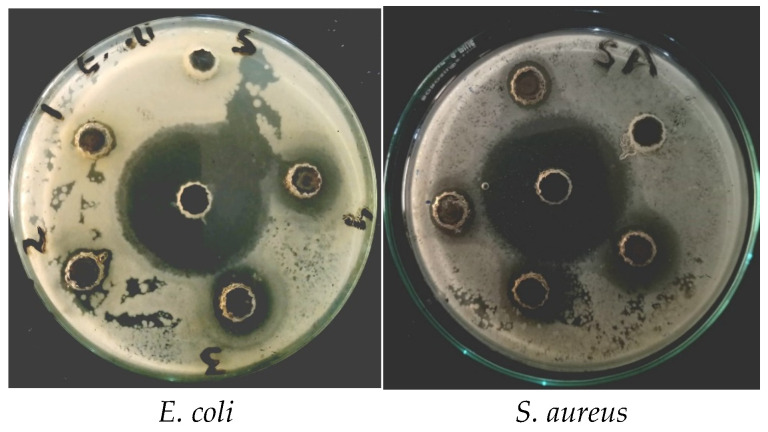
Pictorial representation of bactericidal activity of Ag-CuO NC against *E. coli* and *S. aureus*.
